# Modified Infrahyoid Myocutaneous Flap: A Systematic Review of Surgical Techniques

**DOI:** 10.7759/cureus.113268

**Published:** 2026-07-23

**Authors:** Adit Chotipanich, Panote Kasemsuwan

**Affiliations:** 1 Otolaryngology Department, Chonburi Cancer Hospital, Chonburi, THA

**Keywords:** a systematic review, head and neck reconstructions, infrahyoid myocutaneous flap, surgical techniques, technical modifications

## Abstract

The infrahyoid myocutaneous flap (IHF) is used in reconstructive procedures for small- and medium-sized defects of the head and neck. The IHF is a versatile and technically straightforward reconstructive option; however, it has several limitations, including less reliable skin-paddle vascularity, size constraints, and a relatively short pedicle length. Accordingly, modifications aimed at improving its reliability and versatility have been actively explored. This systematic review aimed to identify available modifications of the IHF, with a focus on surgical technique. A Preferred Reporting Items for Systematic Reviews and Meta-Analyses (PRISMA)-guided search from 1986 to 2025 identified 22 studies encompassing 728 patients. The modifications were categorized into five groups: incision modification, pedicle modification, superficial vein preservation, inclusion of the thyroid gland in the flap, and combination with a microvascular procedure. Among these groups, pedicle modification by periosteal release of the strap muscles from the hyoid was the most commonly used technique. These modifications can also be combined. The pooled estimates for overall flap loss with periosteal release of the strap muscles from the hyoid and vein preservation were 1.66% and 1.62%, respectively. The surgical techniques in each group are highlighted and discussed in this review. Overall, reconstructions using a modified IHF showed improved reliability and may broaden the clinical applications of this flap. However, conclusions regarding the ideal modification cannot be drawn because comparative studies are lacking.

## Introduction and background

In 1986, Wang first described the infrahyoid myocutaneous flap (IHF) for reconstruction in the head and neck region [1]. The IHF is versatile and relatively easy to perform, and many surgeons consider it an alternative to free flaps for medium-sized defects. The advantages of the IHF include good color match, access to the donor site within the same operative field, minimal donor-site morbidity, primary donor-site closure, and appropriate flap thickness for oral defects.

However, the traditional IHF technique has several disadvantages and limitations. Reconstructions using the IHF have frequently encountered problems related to skin-paddle reliability. The IHF is thin and pliable, with a typical skin island averaging 7 × 4 cm. Therefore, the IHF is not suitable for extensive defects that require larger, bulkier flaps. In the traditional technique, the skin paddle is limited to a vertical orientation below the hyoid bone. The IHF also has a relatively short pedicle compared with microvascular free flaps [1-3].

To improve the reliability and versatility of the IHF, modifications to the traditional technique may be beneficial, and several authors have reported favorable outcomes with modified techniques. However, conclusions regarding the optimal surgical technique remain inconclusive because most studies are retrospective and have small sample sizes.

This review aimed to examine and summarize the existing literature on definitions and classifications of modifications for IHF reconstruction. The surgical techniques and flap viability associated with each technique were reviewed.

## Review

Methods

This systematic review was performed in accordance with the Preferred Reporting Items for Systematic Reviews and Meta-Analyses (PRISMA) statement [4]. The review question was formulated using a population, intervention, comparison, and outcome (PICO) framework. The population of interest included patients who underwent IHF reconstruction after tumor resection. The intervention of interest was surgical technique modifications. The primary outcome was flap viability. Flap viability was categorized into three groups: normal, partial (or skin), and total flap loss. The comparator was IHF reconstruction using the traditional technique.

Search Strategy

A search of databases from 1986 to 2025 was undertaken. Two authors independently searched PubMed and Google Scholar for relevant studies using the free-text term “infrahyoid myocutaneous flap.” Hand searches of the reference lists of the included articles were also performed. The literature search and study selection were completed in May 2026.

Inclusion and Exclusion Criteria

Studies were included if they met the following inclusion criteria: (1) the study reported reconstruction using IHF, (2) the surgical technique was described in detail, and (3) the surgery included at least one step that differed from the technique reported by Wang et al. in 1986 (the traditional technique). Exclusion criteria were as follows: (1) a non-English abstract and (2) narrative or systematic reviews.

The key steps of the traditional technique were as follows: (1) The incision is T-shaped. (2) The skin paddle's upper boundary is situated beneath the hyoid bone. (3) The sternohyoid and sternothyroid muscles are cut just above the suprasternal notch. The lower border can be extended below the suprasternal notch if required. (4) The anterior jugular vein is cut and ligated. (5) The IHF is separated from the thyroid gland just above the surface of the capsule. (6) The thyroid and posterior branches of the superior thyroid are ligated, leaving the main trunk of the superior thyroid vessels attached to the flap. (7) The feeding vessels are cleaned from adjacent fascia. (8) The sternothyroid muscle is detached from the thyroid cartilage, and the cricothyroid artery is ligated. (9) The superior laryngeal nerve and ansa cervicalis were preserved. (10) The hyoid insertion of the sternohyoid muscle is divided.

Study Selection and Data Extraction

The retrieved studies underwent title and abstract screening based on the predefined eligibility criteria. Full-text articles were subsequently assessed for inclusion in the review. Studies were included once consensus was reached between the two authors. Data extracted from eligible studies included author details, publication year, study design, sample size, patient characteristics, surgical techniques, flap complications, and key findings. Any uncertainties regarding study eligibility and data extraction were resolved through discussion between the two authors.

Quality Assessment

In this study, we used the Joanna Briggs Institute (JBI) critical appraisal checklist to assess the quality of case reports [5], case series [6], and cohort studies [7]. Studies with scores below 50% were excluded to ensure the reliability of the included research and the credibility of the results.

Statistical Analysis

Pooled analyses of flap loss rates were performed using RevMan software version 5.4 (The Cochrane Collaboration, London, United Kingdom). A fixed-effects model with a 95% confidence interval (CI) was used. Fisher’s exact test was used for comparing categorical variables between the studied groups. Statistical significance was set at p ≤ 0.05.

Results

**Figure 1:**
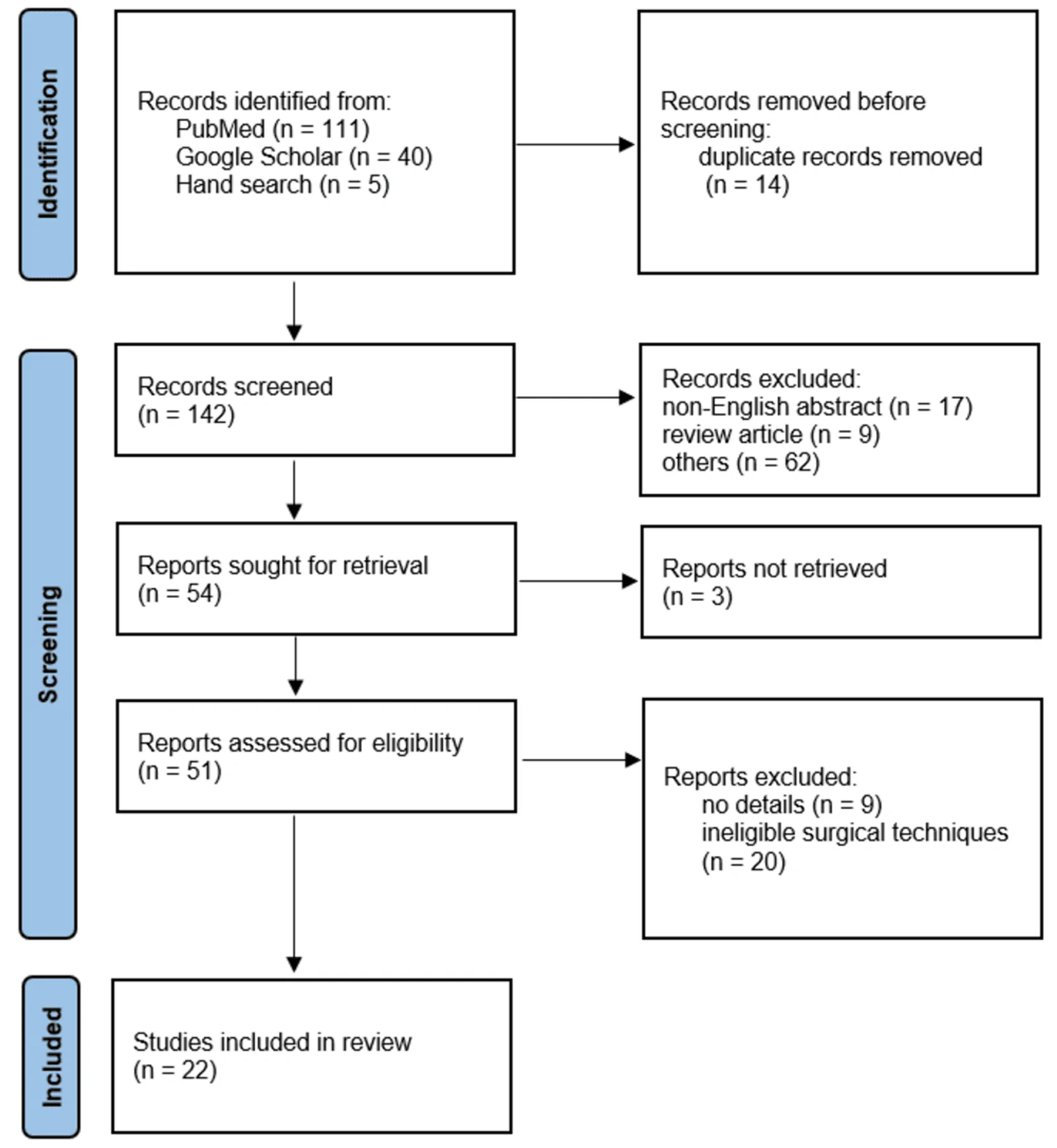
PRISMA 2020 flow diagram PRISMA: Preferred Reporting Items for Systematic Reviews and Meta-Analyses

Figure 1 depicts the PRISMA flow diagram. From 1986 to 2025, a total of 22 studies met the inclusion criteria. Of these, seven were retrospective studies, and the others were case reports or case series [8-29].

The first modification was introduced by Dolivet et al. in 2005, nearly 20 years after the first description of the IHF. The failure rate of this new modification was only 1.1%, which was lower than the 10-47% reported with the traditional technique [1,30-33]. Because of its lower failure rate, Dolivet’s modification has become widely adopted.

Table 1 summarizes the technical modifications and flap loss rates of the included studies. The overall flap failure rates ranged from 0% to 20%. Table 2 shows the categorization of flap modifications. Table 3 summarizes the studies with a comparison between techniques. From seven retrospective studies, two were excluded from the analysis because of a lack of technical detail.

**Table 1:** Summary of studies included in the review * Number of cases with modified techniques

Study	Study design	Cases*	Partial flap loss	Total flap loss	Summary of the modification
Dolivet et al. (2005) [8]	Retrospective	91	1	0	Periosteal release of the strap muscles from the hyoid; S-shaped skin incision
Deganello et al. (2007) [9]	Case series	13	0	0	Preservation of a connection between the superficial and median cervical fasciae around the pedicle
Ricard et al. (2009) [10]	Retrospective	276	22	0	Horizontal flap design
Masic et al. (2012) [11]	Case report	1	0	0	Combination with a microvascular procedure
Deganello et al. (2012) [12]	Retrospective	4	0	0	Preservation of muscular attachments to the hyoid bone
Masuda et al. (2012) [13]	Case report	1	0	0	Modification for trachea reconstruction
Mirghani et al. (2012) [14]	Case series	32	4	0	Dolivet’s modification with preservation of superficial venous drainage
Ouyang et al. (2013) [15]	Retrospective	7	0	0	Preservation of superficial venous drainage
Peng et al. (2013) [16]	Retrospective	13	0	0	Dolivet’s modification with preservation of the anterior jugular vein
Infante-Cossio et al. (2016) [17]	Case series	6	1	0	Dolivet’s modification
Verbruggen et al. (2017) [18]	Case report	1	0	0	Horizontal flap design
Varma et al. (2019) [19]	Case series	9	1	0	Dolivet’s and Deganello’s modifications
Yan et al. (2020) [20]	Case series	10	0	0	Modification for reconstruction after vertical hemicricolaryngopharyngectomy
Jafari et al. (2021) [21]	Retrospective	55	5	1	Inclusion of the superior half of the ipsilateral thyroid gland
Lyu et al. (2021) [22]	Case series	14	1	1	Preservation of anterior jugular vein
Li et al. (2021) [23]	Case series	18	0	1	Modification for voice-tube reconstruction after total laryngectomy
Nguyen et al. (2021) [24]	Case series	103	1	0	Dolivet’s and Deganello’s modifications with preservation of superficial venous drainage
Ranjan et al. (2022) [25]	Case series	10	1	1	Preservation of the anterior jugular vein
Sami et al. (2023) [26]	Case series	12	2	0	Dolivet’s modification
Lu et al. (2024) [27]	Case series	20	0	0	Further modification for voice-tube reconstruction after total laryngectomy
Bagherihagh and Ansari (2025) [28]	Case series	6	0	0	Inclusion of the ipsilateral thyroid gland in the flap and preservation of the anterior jugular vein
Chotipanich (2025) [29]	Retrospective	26	4	0	Preservation of muscular attachments to the hyoid bone

**Table 2:** Categorization of surgical modifications

Aim	Focus	Technique
Reliability	Flap pedicle	(1) Periosteal release of the strap muscles from the hyoid [8]. (2) Preservation of a connection between the superficial and median cervical fasciae around the pedicle [9]. (3) Preservation of muscular attachments to the hyoid bone [29]
	Venous drainage	(1) Preservation of the anterior jugular vein [16,22,25,28]. (2) Preservation of accessory superficial venous drainage [14,15,24]
	Perforator	Inclusion of the thyroid gland in the flap [21]
Versatility	Midline-crossing Defects	Horizontal flap design [10]
	Irregular-shaped defects	Individualized design of skin paddle [13,20,23,27]
	Large defect	Increase bulk by including the thyroid gland in the flap [28]
	Radical neck dissection	Combination with a microvascular procedure [11]
Cosmetic	Incision	(1) S-shaped skin incision [8]. (2) Horizontal flap design [10]

**Table 3:** Summary of comparative studies included in the review

Study	Type	Comparison group		Modification		p-value
		Technique	Overall flap loss	Technique	Overall flap loss	
Dolivet et al. (2005) [8]	Cohort Study	Traditional technique	11.5% (7/61)	Dolivet’s modification	1.1% (1/91)	<0.01
Ouyang et al. (2013) [15]	Cohort Study	Non-preservation of the superficial vein	50% (2/4)	Preservation of the platysma muscle branch	0 (0/7)	0.11
Peng et al. (2013) [16]	Cohort Study	Non-preservation of the superficial vein	57.1% (4/7)	Preservation of the anterior jugular vein	0 (0/13)	<0.01
Nguyen et al. (2021) [24]	Cohort Study	Non-preservation of the superficial vein	11.1% (1/9)	Preservation of the superficial vein	0.97% (1/103)	0.15
Chotipanich (2025) [29]	Cohort Study	Traditional technique	32.4% (11/34)	Preservation of muscular attachments to the hyoid bone	15.4% (4/26)	0.23

All the eligible studies had a low-to-moderate risk of bias. Table 4 shows risk of bias for case reports assessed by JBI critical appraisal tools [5]. Risk of bias assessments for case series and cohort studies are shown in Table 5 and Table 6, respectively [6,7].

**Table 4:** Risk of bias for case reports assessed by the JBI critical appraisal tools Q1: Were patient’s demographic characteristics clearly described? Q2: Was the patient’s history clearly described and presented as a timeline? Q3: Was the current clinical condition of the patient on presentation clearly described? Q4: Were diagnostic tests or assessment methods and the results clearly described? Q5: Was the intervention(s) or treatment procedure(s) clearly described? Q6: Was the post-intervention clinical condition clearly described? Q7: Were adverse events (harms) or unanticipated events identified and described? Q8: Does the case report provide takeaway lessons? Y: yes, N: no or unclear, NA: not applicable, JBI: Joanna Briggs Institute

Study	Q1	Q2	Q3	Q4	Q5	Q6	Q7	Q8	Risk
Masic et al. (2012) [11]	Y	N	Y	Y	Y	Y	Y	Y	Low
Masuda et al. (2012) [13]	Y	N	Y	Y	Y	Y	Y	Y	Low
Verbruggen et al. (2017) [18]	Y	N	Y	Y	Y	Y	Y	Y	Low

**Table 5:** Risk of bias for case series assessed by the JBI critical appraisal tools Q1: Were there clear criteria for inclusion in the case series? Q2: Was the condition measured in a standard, reliable way for all participants included in the case series? Q3 Were valid methods used for identification of the condition for all participants included in the case series? Q4: Did the case series have consecutive inclusion of participants? Q5: Did the case series have complete inclusion of participants? Q6: Was there clear reporting of the demographics of the participants in the study? Q7: Was there clear reporting of clinical information of the participants? Q8: Were the outcomes or follow-up results of cases clearly reported? Q9: Was there clear reporting of the presenting site(s)/clinic(s) demographic information? Q10: Was statistical analysis appropriate? Y: yes, N: no or unclear, NA: not applicable, JBI: Joanna Briggs Institute

Study	Q1	Q2	Q3	Q4	Q5	Q6	Q7	Q8	Q9	Q10	Risk
Deganello et al. (2007) [9]	Y	Y	Y	N	N	Y	Y	Y	N	NA	Moderate
Mirghani et al. (2012) [14]	Y	Y	Y	N	N	Y	Y	Y	N	NA	Moderate
Infante-Cossio et al. (2016) [17]	Y	Y	Y	N	N	Y	Y	Y	Y	NA	Low
Varma et al. (2019) [19]	Y	Y	Y	N	N	Y	Y	Y	N	NA	Moderate
Yan et al. (2020) [20]	Y	Y	Y	N	Y	Y	Y	Y	Y	NA	Low
Lyu et al. (2021) [22]	Y	Y	Y	N	N	Y	Y	Y	Y	NA	Low
Li et al. (2021) [23]	Y	Y	Y	N	N	Y	Y	Y	Y	Y	Low
Nguyen et al. (2021) [24]	Y	Y	Y	N	N	Y	Y	Y	N	NA	Moderate
Ranjan et al. (2022) [25]	Y	Y	Y	N	N	Y	Y	Y	N	NA	Moderate
Sami et al. (2023) [26]	Y	Y	Y	N	N	Y	Y	Y	Y	NA	Low
Lu et al. (2024) [27]	Y	Y	Y	N	N	Y	Y	Y	Y	Y	Low
Bagherihagh and Ansari (2025) [28]	Y	Y	Y	N	Y	Y	Y	Y	Y	NA	Low

**Table 6:** Risk of bias for cohort studies assessed by the JBI critical appraisal tools Q1: Were the two groups similar and recruited from the same population? Q2: Were the exposures measured similarly to assign people to both exposed and unexposed groups? Q3: Was the exposure measured in a valid and reliable way? Q4: Were confounding factors identified? Q5: Were strategies to deal with confounding factors stated? Q6: Were the groups/participants free of the outcome at the start of the study (or at the moment of exposure)? Q7: Were the outcomes measured in a valid and reliable way? Q8: Was the follow-up time reported and sufficient to be long enough for outcomes to occur? Q9: Was follow-up complete, and if not, were the reasons for loss to follow-up described and explored? Q10: Were strategies to address incomplete follow-up utilized? Q11: Was appropriate statistical analysis used? Y: yes, N: no or unclear, NA: not applicable, JBI: Joanna Briggs Institute

Study	Q1	Q2	Q3	Q4	Q5	Q6	Q7	Q8	Q9	Q10	Q11	Risk
Dolivet et al. (2005) [8]	Y	Y	Y	N	N	Y	Y	Y	N	N	Y	Low
Ricard et al. (2009) [10]	Y	Y	Y	N	N	Y	Y	Y	N	N	Y	Low
Deganello et al. (2012) [12]	Y	Y	Y	N	N	Y	Y	Y	N	N	Y	Low
Ouyang et al. (2013) [15]	Y	Y	Y	N	N	Y	Y	Y	N	N	NA	Moderate
Peng et al. (2013) [16]	Y	Y	Y	N	N	Y	Y	Y	Y	N	NA	Low
Jafari et al. (2021) [21]	Y	Y	Y	Y	N	Y	Y	Y	Y	N	Y	Low
Chotipanich (2025) [29]	Y	Y	Y	Y	N	Y	Y	Y	N	N	Y	Moderate

Discussion

The modifications of IHF were categorized into five groups: (1) incision modification, (2) pedicle modification, (3) superficial vein preservation, (4) inclusion of the thyroid gland in the flap, and (5) combination with a microvascular procedure.

Incision Modification

**Figure 2:**
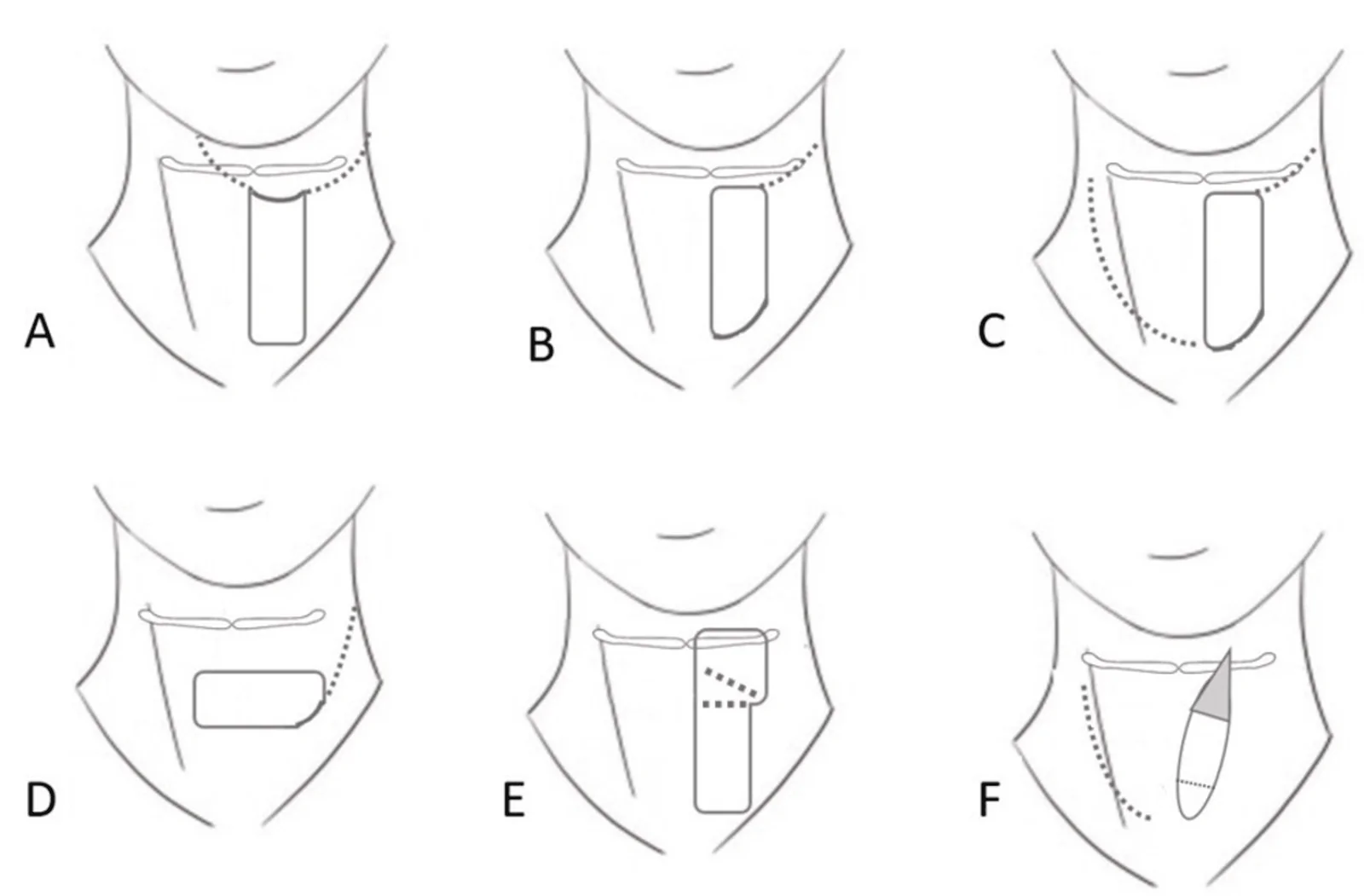
Schematic illustration of the six different types of incisions and flap designs (A) Traditional flap, (B) S-shaped skin incision, (C) S-shaped skin incision for bilateral neck dissection, (D) horizontal flap design, (E) flap design for reconstruction after partial pharyngolaryngectomy, and (F) flap design for voice-tube reconstruction. This image was created by the authors of this study using Paint and PowerPoint 2019 (Microsoft Corp., Redmond, WA, USA). No AI assistance was used.

Several modifications of the incision and skin paddle have been described (Figure 2). The T-shaped incision in the traditional technique (Figure 2A) can provide adequate surgical access for concurrent neck dissection. Still, the three-point junction may compromise wound healing and result in obvious scarring. Modifying the incision to an S-shaped design (Figure 2B-2C) produces better aesthetic results than the original T-shaped incision while maintaining similar surgical accessibility.

The horizontal IHF was introduced in 2008 by Majoufre-Lefebvre (Figure 2D) [10]. This technique has fewer cosmetic sequelae at the donor site when performed with a transverse neck incision, but exposure of the lateral neck is limited. The horizontal, midline position of the skin paddle makes it ideal for reconstruction of the anterior floor of the mouth and tongue. However, a vertically oriented flap has a superior arc of rotation compared with a horizontal flap, allowing reconstruction to reach the upper part of the oral cavity [34].

Use of the traditional IHF for reconstruction after partial pharyngolaryngectomy is less common because of dimensional limitations. These defects require three-dimensional reconstruction to rebuild a new larynx and pyriform sinus. In 2019, Yan et al. introduced a specially designed IHF. This modified IHF was divided into upper, middle, and lower segments (Figure 2E), which were used to reconstruct defects of the laryngotracheal cavity, aryepiglottic fold, and pyriform sinus and lateral pharyngeal wall, respectively [20]. Another design feature in Yan’s modification that departed from the traditional approach was extension of the superior border to 2 cm above the hyoid bone. This might increase the risk of vascular compromise; however, none of the patients in the study experienced flap complications.

Li et al. (2021) first demonstrated the effectiveness of the IHF for reconstructing the pharyngotracheal voice tube in patients who underwent total laryngectomy [23,27]. Li’s flap technique was individually customized from the side opposite the neck dissection, with flap sizes ranging from 1 × 6 to 2 × 8 cm (Figure 2F). The upper and lower portions were sutured upward to the pharyngeal cavity and downward to the trachea, respectively. The middle part of the skin flap was anastomosed together or sutured to the pharyngeal and esophageal serosal layers to create a pronunciation tube.

Irregularly shaped defects after resection of head and neck tumors are not uncommon. The IHF is a versatile flap that can be tailored to specific reconstructive requirements. However, placement of the skin island must include the cricothyroid region [14,34]. The blood supply to the infrahyoid muscles is segmental, and the superior thyroid artery supplies the area above the cricoid ring [35]. Positioning the flap too low can lead to insufficient circulation.

Pedicle Modification

Most authors have acknowledged that the main problems with the traditional technique are related to insufficient venous drainage. In the traditional technique, the skin paddle is nourished by the superior thyroid vessels through perforators of the infrahyoid muscles. Venous drainage of the skin via superficial veins is eliminated, leaving the main venous drainage to depend solely on perforators from the strap muscles. The perforators could be easily damaged by slight shearing or pressure during surgery, making the flap vulnerable to skin necrosis [2,3].

**Figure 3:**
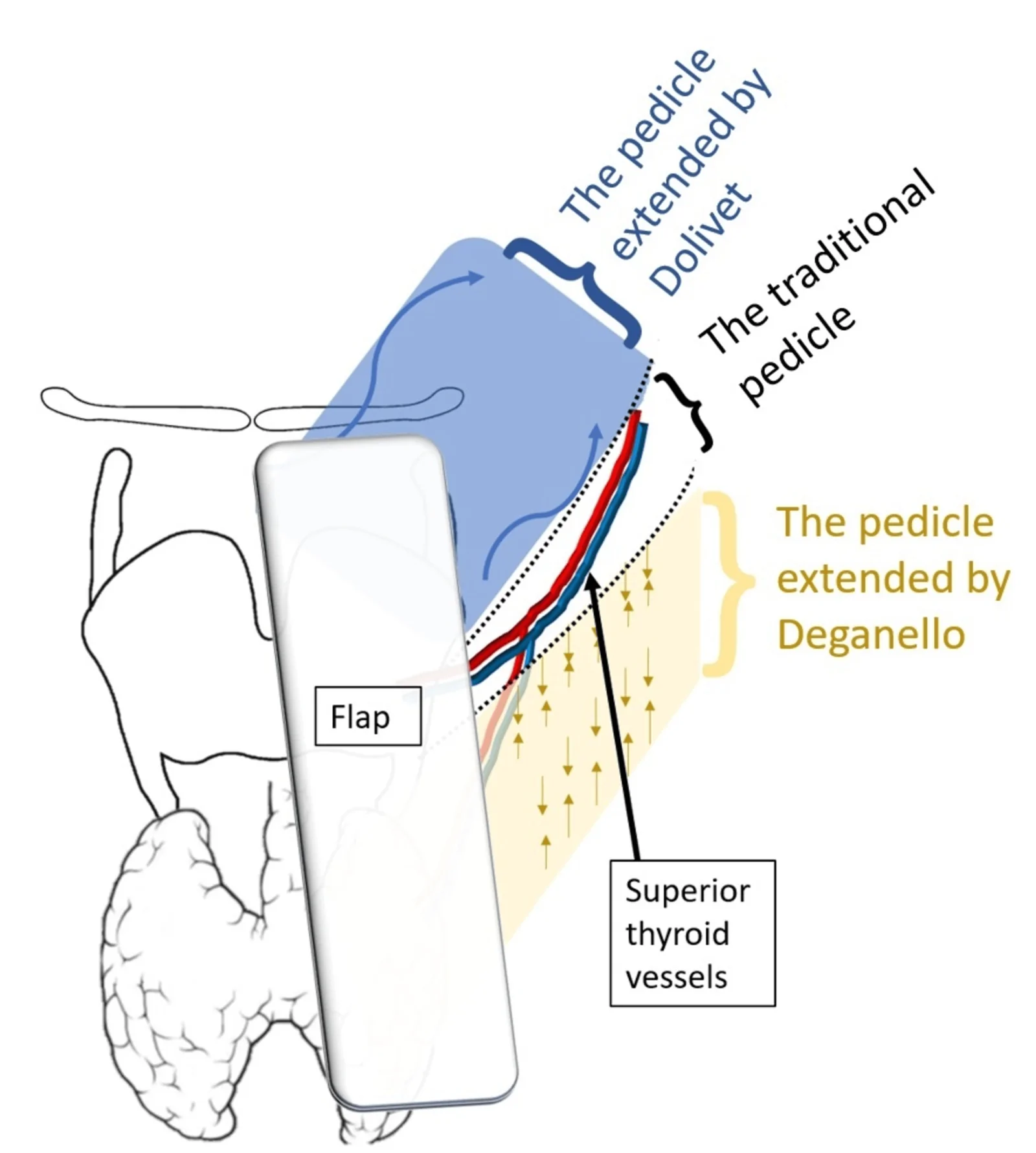
Schematic illustration of the pedicle modifications The black dotted lines indicate the traditional pedicle, which includes only thyroid vessels and ansa cervicalis. (The ansa cervicalis is not shown in the figure.) The blue area indicates the pedicle extended by Dolivet’s modification. The blue arrows represent increased venous drainage towards the digastric triangle network and mylohyoid muscle. The yellow area indicates further pedicle widening by Deganello. The yellow arrows represent increased connection between the superficial and median cervical fasciae. This image was created by the authors of this study using Paint and PowerPoint 2019 (Microsoft Corp., Redmond, WA, USA). No AI assistance was used.

In the traditional technique, the pedicle is composed of the superior thyroid vessels and ansa cervicalis, while the fasciae around the feeding vessels are trimmed (Figure 3). Dolivet’s modification (2004) improves venous drainage towards the digastric triangle network and mylohyoid muscle by periosteal release of the strap muscles from the hyoid [8]. This technique became a hallmark for later modifications. Across eight studies using Dolivet’s modification, the pooled estimate for overall flap loss was 1.66% (95%CI = 0.21-3.12) [8,9,14,16,17,19,24,26].

Deganello et al. suggested a further modification by preserving a greater amount of tissue around the pedicle, resulting in increased communication between the superficial and middle fasciae [9]. These fascial connections are important because they may facilitate microvascular venous return toward the median cervical fascia and protect the superior thyroid vein from twisting or kinking.

Another pedicle modification technique involves preserving the muscular insertion to the hyoid bone. This may improve venous return in a similar manner to Dolivet’s modification. In this modification, the skin paddle is positioned slightly lower than that of the traditional approach. The hyoid bone serves as the center of flap rotation, while the muscles and soft tissue between the skin paddle and the hyoid bone serve as a flap pedicle. Therefore, it is not required to ligate the thyroid gland’s superior pole or separate the strap muscles from the hyoid bone [29].

The primary disadvantage of preserving muscular attachments is the reduced length of the flap pedicle, which limits the flap’s extension and rotation to the defect site. Thus, its application is suitable for defects in the lower oral cavity.

Superficial Vein Preservation

Venous return from the infrahyoid region is achieved primarily through the anterior jugular veins and secondarily through the superior thyroid veins [35]. During harvesting of a traditional IHF, both the distal and proximal segments of the anterior jugular vein and its tributaries are severed. In many cases, venous drainage through perforators is insufficient, resulting in late flap failure.

**Figure 4:**
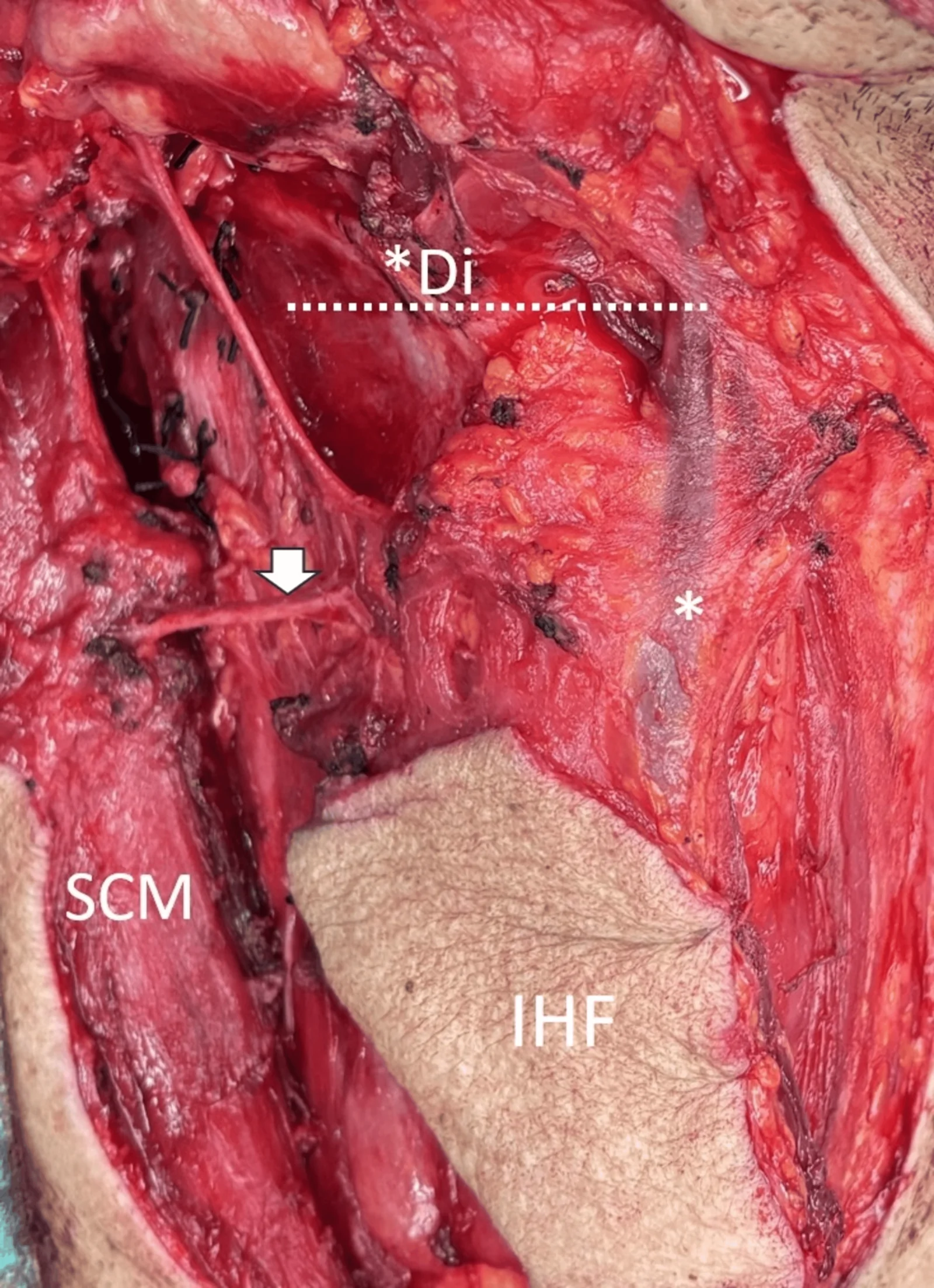
IHF with its superficial veins Preservation of the anterior jugular vein (*) improves venous drainage, with retrograde flow into the external jugular vein. The arrow indicates a small vein that runs alongside the cervical cutaneous nerve into the external jugular vein. IHF: infrahyoid myocutaneous flap (skin paddle), SCM: sternocleidomastoid muscle, white dotted line: hyoid bone level, Di: the intermediate tendon of the digastric muscle. This photograph was taken by Chotipanich A and used with patient consent. No identifiable patient information is disclosed. No AI assistance was used.

Several authors have suggested that preserving superficial venous drainage may result in better venous return from the skin paddle [15,16,22,24,25,28]. Figure 4 shows the superficial veins that can be preserved during flap harvesting. Anterior jugular vein preservation was the most commonly performed technique. Across six studies using the vein preservation technique, the pooled estimate for overall flap loss was 1.62% (95%CI = -0.52-3.75).

A potential disadvantage of vein preservation is that it could increase the risk of undertreating the neck. To avoid compromising oncologic control, vein preservation may not be recommended in patients with clinically involved lymph nodes [3,16].

Inclusion of the Thyroid Gland

In 2021, Jafari et al. modified the IHF by including the superior half of the ipsilateral thyroid lobe, along with its capsule and superior pole above the level of the cricoid [21]. The goal was to use perforator vessels in the upper part of the unilateral thyroid lobe to enhance blood supply to the strap muscles.

Bagherihagh and Ansari further modified the technique by incorporating a unilateral thyroid lobe into the flap [28]. This technique provided additional volume compared with the traditional IHF, enabling repair of larger defects. It should be noted that the attached thyroid tissue may be misinterpreted on postoperative imaging as residual tumor or recurrent disease.

Combining With a Microvascular Procedure

Radical neck dissection is a contraindication for IHF because it removes the internal jugular vein. Masic et al. reported a modified technique in a patient who underwent radical neck dissection. The technique involved combining IHF with a microvascular procedure, specifically venous end-to-end anastomosis to a preservable vein in the contralateral neck [11]. With this modification, radical neck dissection is no longer a contraindication. However, the reliability of this technique cannot be evaluated because no further studies have been published.

Limitations

There are limitations in this review. The first limitation is the inherent selection bias and potential confounding associated with case reports and case series. In most studies, the modified technique was implemented after the surgeon had gained more experience than during the period when the traditional technique was used.

Moreover, there might be some disparities between the studies in the assessment of flap loss because flap viability was assessed by clinical examination. An infrahyoid flap containing only the muscle was excluded in this review [36-38]. Therefore, reconstructions using infrahyoid muscle flaps, such as pharyngeal augmentation, were not examined.

The search strategy of this study also has limitations. Chinese surgeons first pioneered IHF and remain a preferred reconstructive choice in the region. The restriction to English-language studies might have excluded relevant studies in Chinese and other languages. Furthermore, searching only two databases might not provide a thorough summary of the existing literature.

## Conclusions

Although advances in free-flap reconstruction have eclipsed the popularity of the IHF, it remains a useful and highly reliable tool for head and neck reconstruction. One reason is that surgical refinements continue to expand the versatility of the IHF. This review provides surgeons with a comprehensive overview of modified IHF techniques. Failures of the IHF are primarily related to skin-flap necrosis because of the unique venous drainage system of the skin paddle. Thus, most modifications have focused on increasing flap viability by improving venous return. This review also highlights that comparisons between techniques and long-term outcomes remain limited in the literature. Further comparative studies are needed to identify the optimal modification for IHF reconstruction.
